# Prevalence of oral lesions in Langerhans cell histiocytosis: a systematic review and meta-analysis

**DOI:** 10.1007/s10006-026-01586-w

**Published:** 2026-07-03

**Authors:** Mario Dioguardi, Angela Pia Cazzolla, Ciro Guerra, Diego Sovereto, Angelo Martella, Lorenzo Lo Muzio, Khrystyna Zhurakivska, Angela Tisci, Roberto Lovero, Vincenzo Brescia, Giuseppe Troiano, Andrea Ballini

**Affiliations:** 1 Department of Clinical and Experimental Medicine, University of Foggia, Via Rovelli 50, Foggia, 71122 Italy; 2 DataLab, Department of Engineering for Innovation, University of Salento, Lecce, Italy; 3 Department of Medicine and Surgery, University LUM “Giuseppe Degennaro”, Casamasssima, Bari, Italy; 4 Clinical Pathology Unit, AOU Policlinico Consorziale di Bari-Ospedale Giovanni XXIII, Bari, 70124 Italy

**Keywords:** Langerhans Cell Histiocytosis, Mandible, Letterer-Siwe disease, Hand-Schüller-Christian disease, Eosinophilic granuloma, Oral

## Abstract

**Background:**

Histiocytoses are rare diseases and represent a heterogeneous group of pathologies with variable clinical features, determined by the tissue accumulation of cells of presumed dendritic or macrophage origin. Oral manifestations may constitute an early clinical sign and sometimes the only initial evidence of the pathology. The aim of this systematic review with meta-analysis was to investigate the prevalence of oral manifestations in patients affected by LCH, describing their clinical and histopathological characteristics.

**Methods:**

A systematic search was conducted on three major databases (PubMed, Scopus and ScienceDirect) and on Google Scholar grey literature. Relevant articles were selected based on inclusion and exclusion criteria, following the guidelines of the Cochrane handbook. The primary outcome was the prevalence of oral manifestations, calculated as the proportion of affected patients compared to the total cohort in each study. A fixed-effects meta-analysis was performed. A sensitivity analysis was also performed excluding studies with high heterogeneity and a correlation analysis between the mean age at diagnosis and the prevalence of oral lesions, weighted by sample size.

**Results:**

16 studies were included for a total of 2,174 patients. The aggregate prevalence of oral manifestations was 14.86% (95% CI: 11.80–18.58%), reduced to 10.57% in sensitivity analysis. The most frequently involved sites were the mandible and the gums, with osteolytic, ulcerative or periodontal lesions. The weighted correlation analysis between age and prevalence showed a very weak and non-significant correlation (r = 0.007, p = 0.991; Spearman ρ = − 0.258, p = 0.734), confirmed even after the exclusion of outliers.

**Conclusions:**

Oral manifestations represent a relevant clinical component in LCH and may constitute an early diagnostic sign. However, the prevalence varies widely between studies. No significant correlation was observed between age at diagnosis and the presence of oral manifestations. However, the interpretation of these findings is limited by the predominance of retrospective studies and the overall moderate methodological quality of the available evidence. Therefore, further standardized prospective studies are needed to better clarify the clinical impact of oral lesions in LCH.

**Supplementary Information:**

The online version contains supplementary material available at 10.1007/s10006-026-01586-w.

## Introduction

Histiocytoses are rare disorders and represent a heterogeneous group of diseases with varied clinical characteristics determined by the tissue accumulation of cells of presumed dendritic or macrophage origin. These can range from forms limited to the skin to multi-organ diseases, with clinical courses that can be benign or, in some cases, progressive or fulminant, potentially fatal [1].

Approximately 100 different subtypes of histiocytosis have been described. Some forms are of neoplastic origin, such as Langerhans cell histiocytosis (LCH), derived from bone marrow-derived monocytes, as well as Erdheim-Chester disease [2] and the disseminated or extracutaneous form of juvenile xanthogranuloma [3]. For other histiocytoses, a reactive, non-neoplastic origin is suggested [4].

In 1987, histiocytoses were initially classified into three groups: Langerhans cell (LC) or non-LC-related, and malignant histiocytoses (MH) [5].

In 2016, Emile et al. proposed a revised classification that divided histiocytoses into five groups (L-Group, C-Group, R-Group, M-Group, H-Group) based on clinical, histological, and radiological characteristics [6]. This classification is widely accepted today, though it remains in continuous evolution and update.

Histiocytoses that present with mucocutaneous manifestations belong to the Langerhans group (L-group), Langerhans cell histiocytosis (LCH), the group of cutaneous and mucocutaneous histiocytoses identified as the C-group, which includes juvenile xanthogranuloma (JXG), necrobiotic xanthogranuloma (NXG) [7], and multicentric reticulohistiocytosis (MCRH) [6], and the R-group, with RDD and cutaneous RDD (Table 1) [8].

**Table 1:** representing the main forms of histiocytosis that also present with mucocutaneous involvement

Group	Subtype	Description
Group-L	Langerhans Cell Histiocytosis (LCH)	Letterer-Siwe disease, Hand-Schüller-Christian disease, Eosinophilic granuloma
	Erdheim-Chester disease	
Group-C	Juvenile Xanthogranuloma (JXG)	Xanthogranulomas, Benign Cephalic Histiocytosis, Generalised eruptive histiocytoma
	Necrobiotic Xanthogranuloma	Non-xanthogranuloma
	Multicentric Reticulohistiocytosis (MCRH)	
Group-R	Cutaneous Rosai-Dorfman Disease (RDD)	Non-xanthogranuloma
	Rosai-Dorfman Disease (RDD)	

### Main clinical features of histiocytoses with cutaneous and mucosal manifestations

Langerhans Cell Histiocytosis (LCH) primarily affects children under 10 years of age, although approximately 30% of cases occur in adults. Although cutaneous manifestations are observed in about half of patients, skin-limited disease is relatively rare in adults, accounting for only 2% of cases in large studies. When LCH is monosystemic, bone involvement is the most common, followed by skin, lymph nodes, and lungs [9].

The cutaneous manifestation typically resembles eczematous lesions, sometimes resembling seborrheic dermatitis. Other cutaneous manifestations may be psoriasiform or acneiform, and there can be nodular and ulcerated forms [10].

Among the most common forms of non-Langerhans cell histiocytosis, Juvenile Xanthogranuloma (JXG) is usually benign, although it has been associated with the onset of juvenile myelomonocytic leukaemia [11]. The eye is the most common site of extracutaneous manifestation, predominantly affecting individuals within the first year of life, and is 10 times more frequent in those with fair skin compared to those with darker skin [12].

The primary cutaneous manifestation is located on the head, neck, and trunk, typically presenting as a solitary yellowish erythematous nodule. There are two clinical forms: a small nodular form (1–2 mm) and a larger nodular form (10–20 mm) [12].

Necrobiotic Xanthogranuloma (NXG) is a rare granulomatous disease with progressive multisystem involvement, characterised by monoclonal gammopathy in 80% of cases. It is characterised by destructive cutaneous and subcutaneous lesions, with ocular manifestations being common [13].

The lesions are typically asymptomatic yellowish papules and nodules, measuring 0.5–2 cm, which tend to coalesce into indurated plaques with telangiectasias that may ulcerate and subsequently heal with scarring, predisposing to the development of additional lesions. Their distribution is typically on the trunk, extremities, and periorbital locations [14].

Multicentric Reticulohistiocytosis (MCRH) predominantly affects adults, with a higher prevalence in females aged between 50 and 60 years. The clinical picture involves widespread cutaneous involvement, destructive arthritis, and fever [15].

Lesions are typically erythematous or brown papules and nodules, ranging from a few millimetres to 2 cm, and are located on the extremities and acral areas. Classic findings include periungual papules described as “coral bead” (pathognomonic). About 50% of patients also present with nodules on the oral or nasal mucosa [16].

Rosai-Dorfman Disease (RDD), also known as sinus histiocytosis with massive lymphadenopathy, primarily affects children and young adults, with a higher prevalence in males and individuals of African descent in the systemic form. Cutaneous RDD, on the other hand, predominantly affects elderly Asian women, with a higher incidence among Asians and Caucasians. The lesion type typically consists of papules, nodules, or coalescing plaques with surrounding satellite lesions, commonly located on the trunk, head, or neck [17].

The considerable heterogeneity of conditions classified as histiocytoses, along with the variability of their oral manifestations, raises questions regarding the actual prevalence of oral and jawbone lesions associated with these diseases. Prevalence data in the literature are highly inconsistent, ranging from reports indicating rates above 80% (Sedano et al., 1969 [18]) to studies reporting values slightly over 10% (Hartman, 1980 [19]) or even 0% (Cochrane et al., 2003 [20]). Moreover, the nature of these lesions, their clinical presentation, and their anatomical distribution remain insufficiently defined. In many cases, intraoral lesions caused by histiocytosis may be easily mistaken for neoplastic, proliferative, or inflammatory conditions, or for other more common pathologies.

In this review, in order to reduce heterogeneity in data and interpretation, we have focused exclusively on lesions classified as Langerhans Cell Histiocytosis (LCH) belonging to Group L (Table 1). The aim is to provide a reliable estimate of the prevalence of oral lesions in LCH, thereby offering clinicians, particularly general dentists and paediatric dentists, evidence-based information on the likelihood of oral involvement at the time of diagnosis, which typically occurs during childhood. Furthermore, the review aims to provide relevant insights into the common anatomical locations of these lesions within the oral cavity.

## Materials and methods

### Protocol and registration

This systematic review was preceded by a preliminary phase involving an in-depth analysis of the literature concerning oral lesions and manifestations of histiocytoses. Based on this preliminary assessment, we conducted a systematic review in accordance with the PRISMA (Preferred Reporting Items for Systematic Reviews and Meta-Analyses) guidelines [21]. Accordingly, the processes of literature search, study selection, and data extraction were conducted according to the recommendations outlined in the Cochrane Handbook. Furthermore, the review protocol was submitted and prospectively registered on the PROSPERO platform (International Prospective Register of Systematic Reviews) under the registration number CRD420251068995 prior to the commencement of study selection.

### Eligibility criteria

All clinical and epidemiological studies investigating oral manifestations of histiocytoses were considered potentially eligible. Particular emphasis was placed on studies that specifically examined and reported data on lesions affecting the oral mucosa and jaw bones as oral manifestations of histiocytoses.

The PICO question was formulated as follows: What is the prevalence of oral manifestations of histiocytosis in patients diagnosed with histiocytosis?

- (P) Population: Patients with a diagnosis of histiocytosis.
- (I) Intervention: Oral manifestations of histiocytosis involving the mucosa and jaw bones.
- (C) Comparison: Not applicable.
- (O) Outcome: Prevalence of oral lesions and manifestations in patients diagnosed with histiocytosis.

The inclusion criteria encompassed all reports of clinical trials and epidemiological studies conducted on patients with a confirmed diagnosis of histiocytosis that provided data on the presence of oral and maxillary bone lesions and manifestations.

The decision to include only clinical and epidemiological studies, excluding case reports and case series, was based on the need to obtain prevalence data concerning oral manifestations within a study population representative of a broader cohort of patients with histiocytosis. Such data cannot be reliably derived from isolated case reports or small case series.

The following exclusion criteria were applied:

- Studies or reports corresponding to systematic reviews, scoping reviews, mapping reviews, narrative reviews, case reports, case series, and in silico studies.
- Studies published in languages other than English, or for which a clear and reliable translation was not available or feasible.
- Studies involving fewer than 20 patients.
- Reports considered to be at high risk of bias.
- No exclusion criteria were applied based on the year of publication.

### Sources of information, search strategy and study selection

The search for articles and reports was conducted using online search engines by two independent reviewers, who are also the authors of this manuscript (M.D. and A.T.). Preliminary exclusion criteria included language restrictions: reports lacking at least an abstract in English were excluded using automated tools available within the selected databases.

The search engines and databases used included PubMed, Scopus, and the Cochrane Library. In addition, grey literature was retrieved through Google Scholar, ScienceDirect, and the DANS Archive (Data Station Life Sciences) [22]. To further reduce the risk of publication bias, the reference lists of previous reviews on histiocytosis were also screened. The initial search, including the last update of identified records, was completed on 1 April 2025. A final literature update was conducted on 11 May 2025.

Search terms were selected to capture the widest possible range of studies focused on histiocytosis and its oral manifestations. The following search strategies were employed across the selected databases:

- PubMed:
- Search: *“langerhans cell histiocytosis” OR LCH*.
- Query: *“langerhans cell histiocytosis“[All Fields] OR “LCH“[All Fields]*.
- Scopus:
- TITLE-ABS-KEY(*“langerhans cell histiocytosis”*).
- Cochrane Central Register of Controlled Trials:
- Search term used in Title and Abstract: *“langerhans cell histiocytosis”*.

To ensure transparency, a summary table was included (Table 2) listing the web addresses of the databases, the date of the last search, and the number of records retrieved (excluding grey literature).

**Table 2:** Table showing the addresses of the databases where the records were searched * you must be logged in to Scopus

Research databases or bibliographic databases	Web address	Data	Records
PubMed	https://pubmed.ncbi.nlm.nih.gov/?term=%22langerhans+cell+histiocytosis%22+OR+LCH+&ac=yes&cauthor_id=None&user_filter=&schema=none&page=1&whatsnew=None&show_snippets=on&sort=date&sort_order=desc&format=summary&size=200	15 May 2025	6724
	Search: “langerhans cell histiocytosis” OR LCH Sort by: Most Recent		
Scopus*	https://www.scopus.com/results/results.uri?st1=%22langerhans+cell+histiocytosis%22&st2=&s=TITLE-ABS-KEY%28%22langerhans+cell+histiocytosis%22%29&limit=10&origin=searchbasic&sort=plf-f&src=s&sot=b&sdt=b&sessionSearchId=25cfd5dbdb514a6d1b5ff6aa3ff12aef	15 May 2025	8248
	TITLE-ABS-KEY ( “langerhans cell histiocytosis”)		
Cochrane Central Register of Controlled Trials (CENTRAL)	https://www.cochranelibrary.com/search	15 May 2025	61
	“langerhans cell histiocytosis” in Title Abstract Keyword		

The records identified were imported into EndNote, and duplicates were automatically detected and removed using the software’s “Find Duplicates” function. Any additional duplicates not recognised by the software were manually removed by the reviewers responsible for study selection.

Article selection was performed independently by two reviewers (M.D. and A.P.C.). Initially, they compiled lists of potentially eligible studies and subsequently included them in two separate tables, which were then compared. Potentially eligible studies were identified through title screening, while studies meeting the inclusion criteria were selected following full-text review and analysis.

Reviewer agreement was also assessed, and any discrepancies were resolved by a third reviewer (A.B.).

### Data collection process and data characteristics

The data to be included in the summary tables of the selected studies were defined during the initial development of the review protocol. As with the data assessed during the selection and screening phases, data extraction was performed independently by the two reviewers and subsequently cross-checked to minimise potential inaccuracies. One reviewer then compiled the verified information into a unified table.

Any discrepancies identified during the data extraction process were initially documented and acknowledged. To address such issues, the reviewers engaged in thorough discussions to reconcile differences, clarify misunderstandings, and resolve potential errors. In cases where consensus could not be reached through discussion, unresolved issues were referred to a third reviewer. The latter carefully reviewed the disputed data and provided a final judgement on the accuracy of extraction. This procedure ensured that all conflicts were resolved systematically and transparently, based on the available evidence.

Extracted data included: first author, year of publication, study design, country of origin, number of patients included, type of histiocytosis, number of patients with oral manifestations, type of intraoral lesions and, where reported, their location, mean age at diagnosis, and sex.

### Risk of bias in individual and cross-sectional studies, summary measures and outcomes, publication bias, GRADE

Particular emphasis was placed on assessing the risk of bias using the Appraisal Tool for Cross-Sectional Studies (AXIS tool), which is suitable for both retrospective cross-sectional studies and descriptive cohort studies without a comparison group. This instrument is particularly appropriate for evaluating studies reporting the prevalence of oral lesions in patients with histiocytosis.

Studies identified as being at high risk of bias were excluded from the meta-analysis. As in the other phases of the review process, risk of bias assessment was performed independently by two reviewers (M.D. and A.P.C.), with discrepancies resolved by a third reviewer who acted as adjudicator. The results were subsequently compared and consolidated into a single summary table.

The findings of the review were presented in tables, with aggregate data displayed both numerically and graphically. Graphical representations included forest plots and heterogeneity indices such as Higgins’ I². Between-study bias was assessed visually by examining the overlap of confidence intervals (CIs), and quantified using the I² inconsistency index. An I² value greater than 50% was considered indicative of moderate heterogeneity. In cases of high heterogeneity, sensitivity analyses were planned by excluding studies with poor CI overlap or disproportionately large weights.

For the meta-analysis, specifically evaluating the proportion of patients with oral lesions among the total population with histiocytosis, OpenMeta[Analyst] version 10 (Centre for Evidence-Based Medicine, Brown University) was used.

Publication bias was assessed using a dedicated Python-based application previously developed by the authors [23]. Funnel plot inspection and Egger’s regression test were used to evaluate potential asymmetry among studies. The application was made available as supplementary material.

Heterogeneity between studies was assessed using Higgins I² index and Cochran’s Q test.

The quality of evidence was assessed using the GRADE approach. The results were summarised in a dedicated table.

## Results

### Study selection

The searches conducted across SCOPUS, PubMed, and the Cochrane Central Register of Controlled Trials yielded a total of 15,033 bibliographic records. After the removal of duplicates (4,115 records), 34 potentially eligible articles remained and were subjected to full-text screening. Of these, only 11 articles met the inclusion criteria for the systematic review and meta-analysis.

Additionally, grey literature searches carried out in repositories such as DANS (Data Station Life Sciences) (https://ssh.datastations.nl/), ScienceDirect, Google Scholar, and previous systematic reviews using the keyword “Langerhans cell histiocytosis” identified a further 5 eligible studies for inclusion in the meta-analysis.

**Fig. 1:**
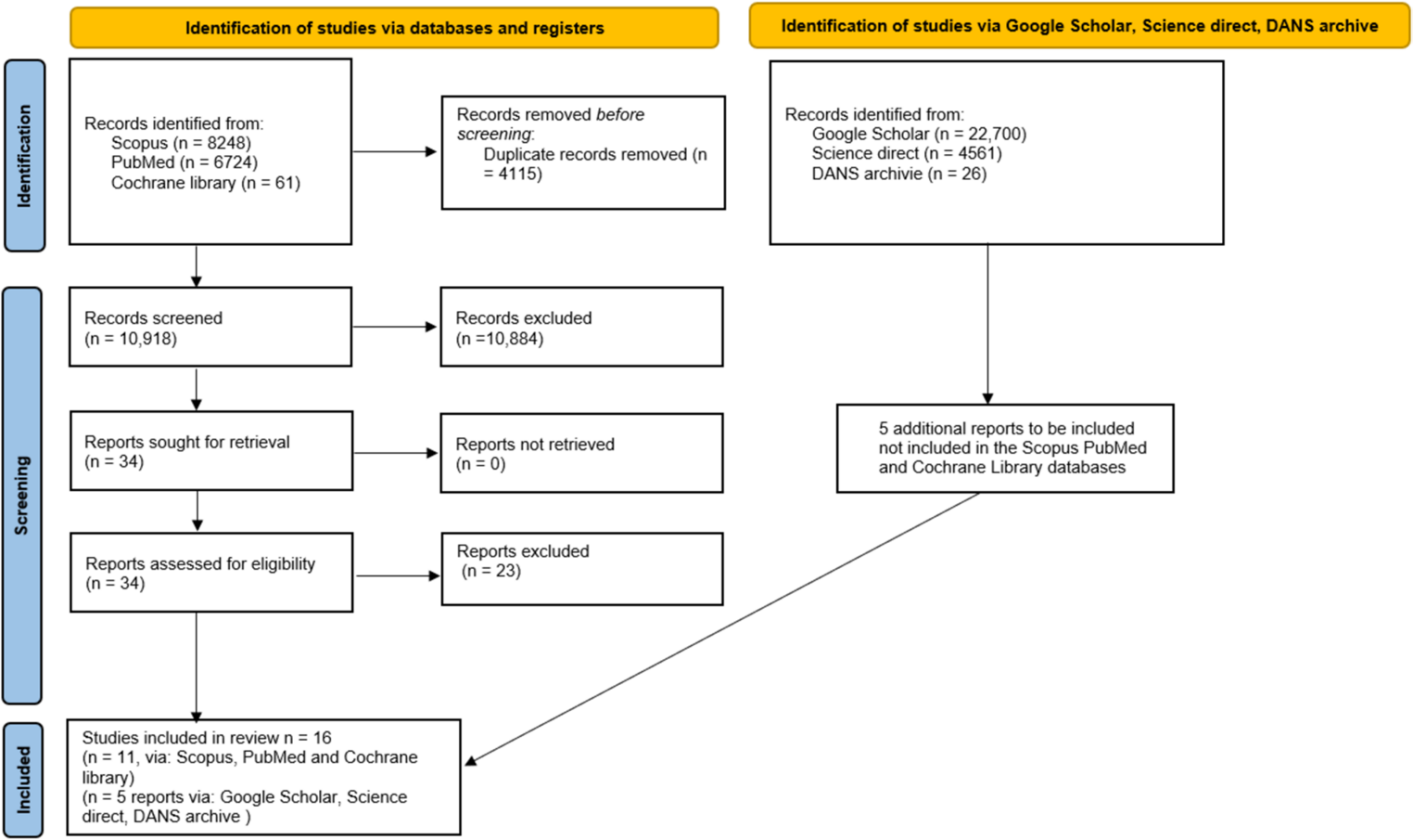
Flow chart of the study selection and inclusion process

The study selection process demonstrated that a sufficient number of studies were available (more than 10) to perform a robust meta-analysis and determine an estimate of the prevalence of oral lesions in patients with histiocytosis. The complete procedure for identification, selection, and inclusion of studies is illustrated in the PRISMA flow diagram shown in Fig. 1.

During this phase, 23 studies were excluded for the following main reasons:

- A total of seven studies did not report or investigate the presence of manifestations involving the oral mucosa or jaw bones (Category A);
- Six studies included only patients with oral manifestations or head and neck involvement, identified retrospectively, without considering the entire cohort of patients diagnosed with histiocytosis (Category B);
- Four studies included a sample size of fewer than 20 patients (Category C);
- Three studies were literature reviews not explicitly identified as such in the title or abstract (Category D);
- One study consisted of clinical guidelines, position papers, or survey-based research (Category E);
- One study was a case series (Category F);
- One study involved only the retrospective analysis of histological samples from the oral cavity (Category G).

The excluded studies, along with their respective reasons for exclusion, are listed in Table 3 and the characteristics of excluded studies were transferred to the Supplementary Materials.

**Table 3:** Excluded Studies and Reasons for Exclusion. Studies excluded after full-text screening with justification based on predefined eligibility criteria. (A): No data on oral manifestations or involvement of jaw bones reported; (B): Only patients with oral manifestations were included, without assessing the full histiocytosis cohort; (C): Sample size fewer than 20 patients; (D): Narrative or non-systematic review, not clearly identified in title or abstract; (E): Clinical guidelines, position papers, or survey-based studies; (F): Case report or case series; (G): Retrospective analysis performed only on histological specimens, without complete clinical data

Data, autor	Country	Type of study	Reasons for exclusion	
McCaffrey and McDonald, 1978 [24]	USA	Retrospective	No data on manifestations on the oral mucosa or jaw bones are reported	A
Jones an Pillsbury, 1984 [25]	USA	Case series	Case series	F
Broadbent et al., 1989 [26]	USA	Clinical Guidelines	Clinical Guidelines on Histiocytosis syndromes in children	E
Hamre et al.,1997 [27]	USA	Retrospective	does not report details of oral manifestations or specific involvement of the oral cavity	A
Wang et al., 2010 [28]	China	Retrospective	The manuscript mainly reports bone involvement of other sites, such as the skull, femur, tibia, and ilium, but does not explicitly mention lesions of the mandible or maxilla	A
Modest et al., 2016 [29]	USA	Retrospective	The manuscript mainly reports bone involvement in temporal bone	A
Neves-Silva et al., 2018 [30]	Brazil	Retrospective	Only patients presenting with oral manifestations were included	B
Nicollas et al., 2010 [31]	France	Retrospective	No cases of mandibular or maxillary involvement have been reported	A
Dagenais et al., 1992 [32]	Canada	Retrospective	Only patients presenting with oral radiografic manifestations were included	B
Mortellaro et al. 2006 [33]	Italy	Retrospective	They only analyzed 8 cases	C
Hicks and Flaitz, 2005 [34]	Usa	Review	Review : the suspected neoformative nature of the LHC	D
Gadner et al., 2001 [35]	Austria	RCT	does not report details of oral manifestations or specific involvement of the oral cavity	A
Bartnick et al. 2002 [36]	Germany	Retrospective	They only analyzed 12 cases	C
Ardekian et al., 1999 [37]	Israel	Retrospective	Only patients presenting with oral radiografic manifestations were included	B
Howarth et al., 1999 [38]	USA	Retrospective	does not report details of oral manifestations or specific involvement of the oral cavity	A
Slater and Swarm, 1980 [39]	USA	Review	Review: analysis of eosinophilic granuloma cases	D
Egeler and Nesbit, 1995 [40]	Netherlands, USA	Review	Review: LHC and other disorders of monocyte-histiocyte	D
Eckardt and Schultze, 2003 [41]	Germany	Retrospective	Only 10 Cases	C
Mínguez et al., 2003 [42]	Spain	Retrospective	Only 10 Cases	C
Lewoczko et al., 2014 [43]	USA	Retrospective	Only patients presenting with Head and Neck manifestations were included	B
Bedran et al., 2018 [44]	Guatemala, Brazil	Retrospective	Only patients presenting with Head and Neck manifestations were included	B
Jalil and Hin-Lau, 2009 [45]	Malaysia	Retrospective	Only patients presenting with oral manifestations were included	B
Atarbashi Moghadam et al., 2015 [46]	Iran	Retrospective	Cases of oral manifestation of LCH were identified retrospectively on SPECIMENS received at the Oral and Maxillofacial Pathology Department.	G

### Data characteristics

The reports included in this review comprise 16 studies published between 1969 and 2023: Pavan et al., 2023 [47], Lin et al., 2023 [48], García et al., 2022 [49], Lee et al., 2014 [50], Annibali et al., 2009 [51], Buchmann et al., 2006 [52], Cochrane et al., 2003 [20], al-Ammar et al., 1999 [53], Quraishi et al., 1995 [54], Irving et al., 1994 [55], Gadner et al., 1994 [56], DiNardo and Wetmore, 1989 [57], Hartam, 1980 [19], Rapidis et al., 1978 [58], Sigala et al., 1972 [59] and Sedano et al., 1969 [18]:

The extracted data are presented in a table. Table 4 summarises the information regarding the first author, year of publication, study design, country of origin, number of patients diagnosed with histiocytosis, sex, anatomical localisation of the lesions, and mean age at diagnosis.

**Table 4:** Characteristics of studies included in the review of oral manifestations in histiocytosis patients

Autor, Data	Country	Type of study	Patient	Age, Me, Ra	Lesion
Pavan et al., 2023 [47]	Argentina	Retrospective, Single Center	95( 41 F 54 M)	0-16y, 2.7 ± 2.9 y	40 (21 bone, 16 bone mucosal, 3 mucosal)
Lin et al., 2023 [48]	Taiwan	Retrospective, Single Center	67 (44d , 21 F 23 M)	11.7y	7 (4 mandibular 3 Maxillar)
García et al., 2022 [49]	Spain	Retrospettive, Single Center	20 (7 F 13 M)	6.5y, (2–12y)	1(mandibular mucocutaneo)
Lee et al., 2014 [50]	Republic of Korea	Retrospective, Single Center	154	4.1y, (0.0–15.3y)	21 (mandibular bone)
Annibali et al., 2009 [51]	Italy	Prospective, Single Center	31 (16 F 15 M)	37.3 y, (18-73y)	12
Buchmann et al., 2006 [52]	USA	Retrospective, Single Center	22 (2 F 12 M)	3.2y, ( 0.8-11y)	1 mandibular, 1 maxillary
Cochrane et al., 2003 [20]	USA	Retrospective, Single Center	21 (8 F 13 M)	5.7y	0
al-Ammar et al., 1999 [53]	Canada	Retrospective, Single Center	20 (7 F 13 M)	5.6 y, (0-14y)	2 (mandibular e maxillary)
Quraishi et al., 1995 [54]	Ireland, UK	Retrospective, Two Center	73 (28 F 45 M)	3-15y	5 mandibular and oral mucosal
Irving et al., 1994 [55]	UK	Retrospective, Two Center	131 (48 F 83 M)	3.2y (0-16y)	13 maxillary, 8 mandibular
Gadner et al., 1994 [56]	Austria, Germany, Netherlands	RCT, multicenter	106 (48 F 58 M)	1.5y, (0.3-14y)	10 gingiva a
DiNardo and Wetmore, 1989 [57]	USA	Retrospective, Single Center	100 (37 F 67 M)	3.8y, (0-22y)	8 mandibular 5 gengival c
Hartam, 1980 [19]	USA	Retrospective, Single Center	1120 (20 F 94 M)	≈ 14.4y, (0-53y)	114
Rapidis et al., 1978 [58]	UK	Retrospective, Single Center	50 (20 F 30 M)	15.3y	27 b
Sigala et al., 1972 [59]	USA	Retrospective, Single Center	50 (26 F 24 M)	≈ 13.5, (1-67y)	18 (gingival and erosion bone)
Sedano et al., 1969 [18]	France, USA	Studio retrospettivo, Two Center	22 (9 F 13 M)	≈ 9,7 1-45y	17 oral mucosa (13 mandibular or maxillary)

^a^ The data refer to 78 patients, not 106 or 199.

^b^ A total of 27 patients are reported as presenting with oral manifestations only.

^c^ The data concerning the gingiva and mandible cannot be summed, as the text does not clarify whether these are distinct patients or whether the same individuals exhibited both manifestations.

^d^ Forty-four patients presented with head and neck manifestations; sex-specific data are available only for this group

**Fig. 2:**
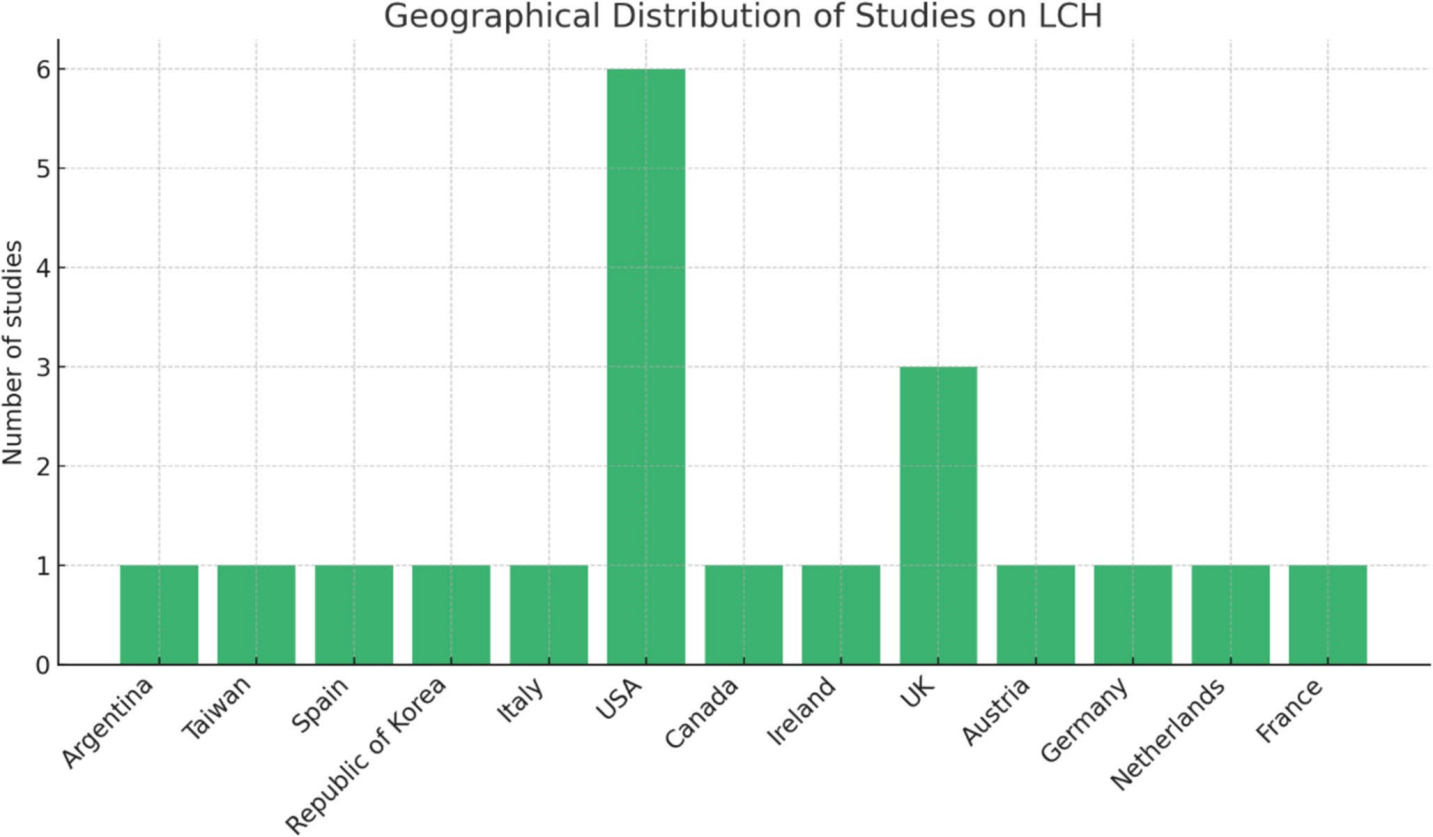
Graph illustrating the geographical distribution of studies on Langerhans Cell Histiocytosis (LCH), based on the country of affiliation of the corresponding authors

The data come from studies conducted in Europe, North America, Asia and South America (Fig. 2), with mainly retrospective observational designs; only 2 studies: Gadner et al., 1994 [56] which represents a multicenter prospective trial and Annibali et al., 2009 [51] with a prospective observational study.

The number of patients varied widely among the studies, ranging from 20 cases reported by García et al., 2022 [49], and al-Ammar et al., 1999 [53], to the 1,120 cases reported by Hartam, 1980 [19], which is also the study with the highest number of oral manifestations of histiocytosis. The study by Pavan et al., 2023 [47], also reported a substantial number of oral manifestations.

The total number of patients with histiocytosis included across all studies was 2,082, of whom just over half (1,120) were included in the report by Hartam, 1980 [19].

In total, 340 female and 574 male patients were identified, while the sex of 1,168 patients was not reported (specifically in Hartam, 1980 [19] and Lee et al., 2014 [50]).

The weighted mean age at diagnosis was approximately 7.6 years, calculated based on the number of patients in each study.

**Fig. 3:**
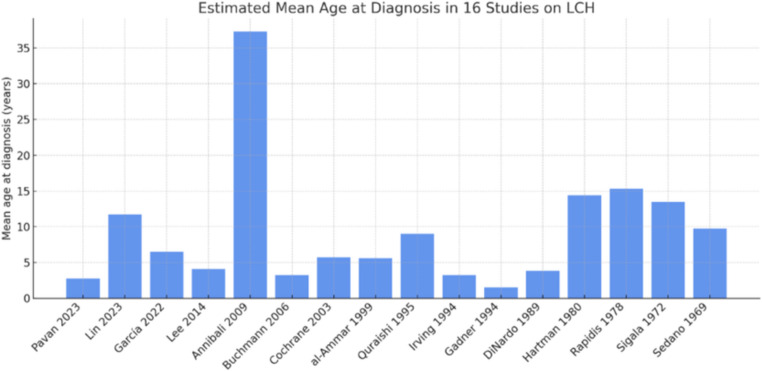
Estimated mean age at diagnosis in 16 studies on Langerhans Cell Histiocytosis (LCH). Bar chart illustrating the variation in mean age at diagnosis across included studies

The average age at diagnosis was strongly influenced by the clinical presentation: studies including patients with disseminated LCH reported lower mean ages (1.5–6 years), whereas those focusing on localised or chronic forms showed diagnoses occurring in adolescence or adulthood (up to 20 years in the study by Hartman) (Fig. 3).

**Fig. 4:**
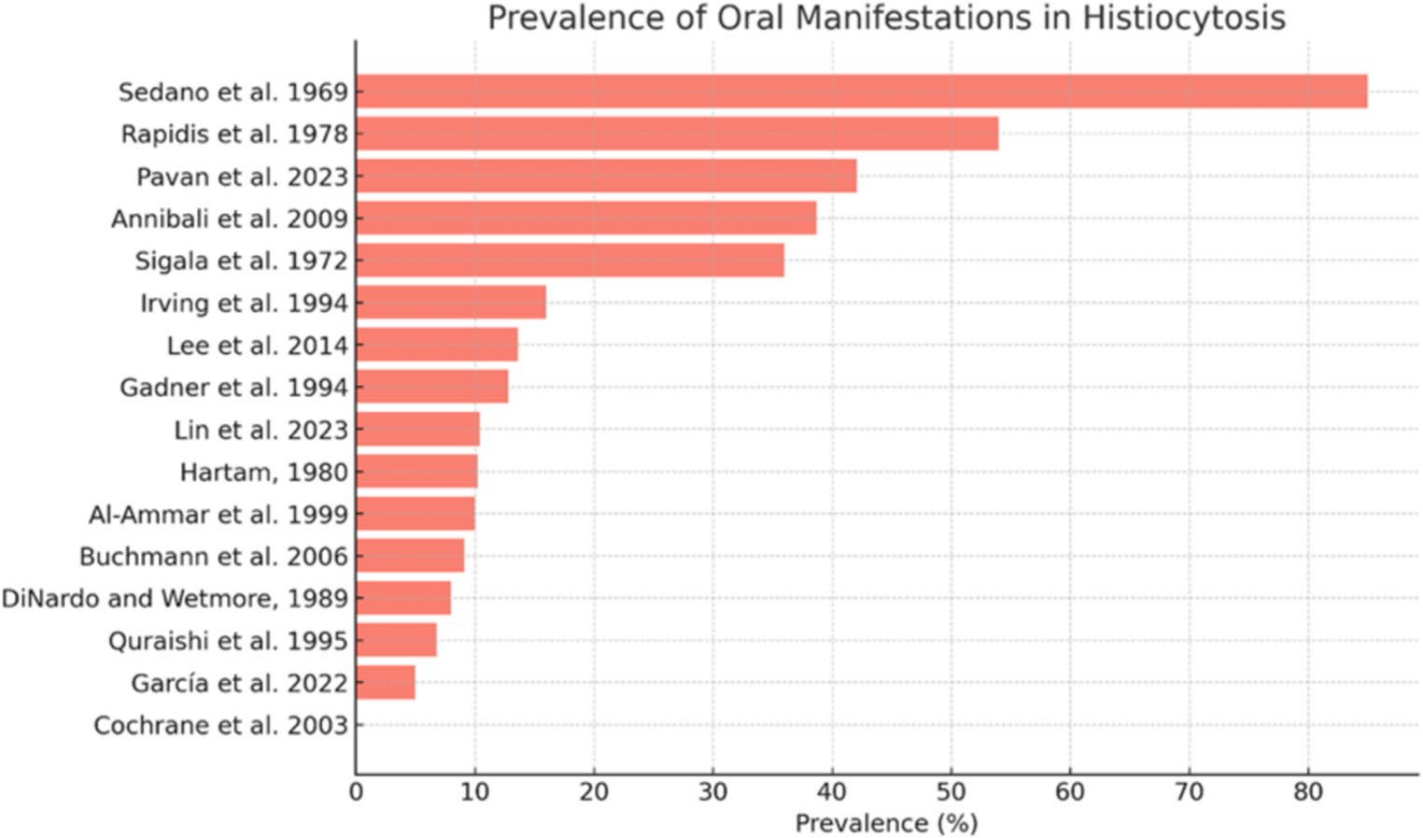
Prevalence of oral manifestations in histiocytosis across included studies; Horizontal bar chart displaying the percentage of patients with oral manifestations reported in each study

**Fig. 5:**
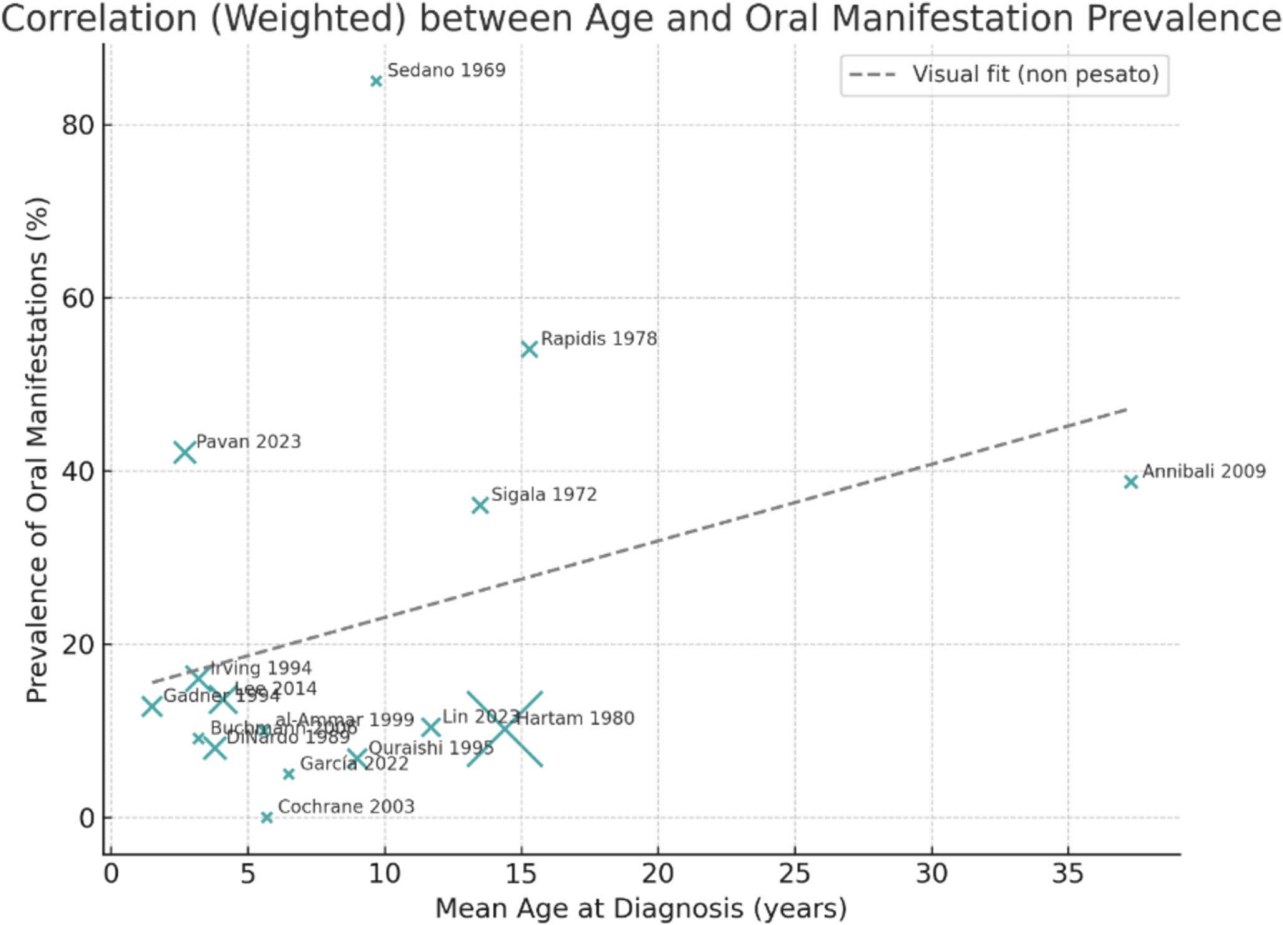
Weighted correlation between mean age at diagnosis and prevalence of oral manifestations in LCH: Scatter plot showing the relationship between average age at diagnosis and oral manifestation prevalence, with bubble size proportional to study sample size. Dashed line represents unweighted visual trend

A correlation was also performed between the prevalence of oral lesions (Fig. 4) in relation to the age of diagnosis (Fig. 5). Both correlation coefficients (Pearson correlation: *r* = 0.337, *p* = 0.202, Spearman correlation: ρ = 0.218, *p* = 0.418) indicate a weak positive trend, i.e. a slight association between older age at diagnosis and higher prevalence of oral lesions. However, the p values are not statistically significant, suggesting that there is not enough evidence to affirm the existence of a linear or monotonic correlation. Furthermore, when the analysis was weighted according to study sample size, no statistically significant association is found between the mean age at diagnosis and the prevalence of oral manifestations (Pearson *r* = 0.007, *p* = 0.991; Spearman ρ = − 0.258, *p* = 0.734). This suggests that age at diagnosis is not a relevant determinant of the presence of oral manifestations in patients with histiocytosis, considering the size of the studies.

**Fig. 6:**
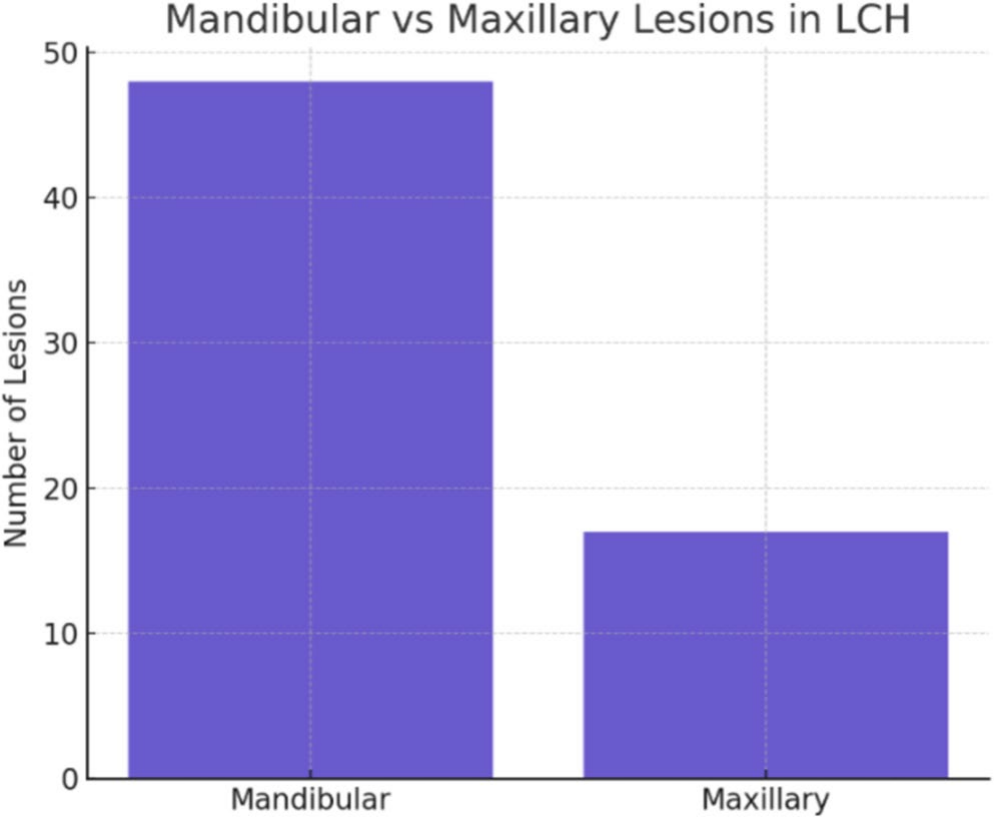
Comparison of Mandibular and Maxillary lesions in patients with Langerhans Cell Histiocytosis (LCH); Bar chart illustrating the number of lesions reported in the mandible versus the maxilla across the included studies

In most studies, the mandible was the most frequently involved oral site, (Fig. 6) Figure takes into account not only bone lesions but also mucosal lesions located above the mucosal bone crest, even if the location is clear : Lin et al., 2023 [48] 4 mandibula 3 Maxillary, García et al., 2022 [49] 1 mandibular, Lee et al., 2014 [50] 21 mandibular, Buchmann et al., 2006 [52] 1 mandibular, 1 maxillary, Quraishi et al., 1995 [54] 5 mandibular, Irving et al., 1994 [55] 13 maxillary, 8 mandibular, DiNardo and Wetmore, 1989 [103] 8 mandibular.

### Risk of bias

**Fig. 7:**
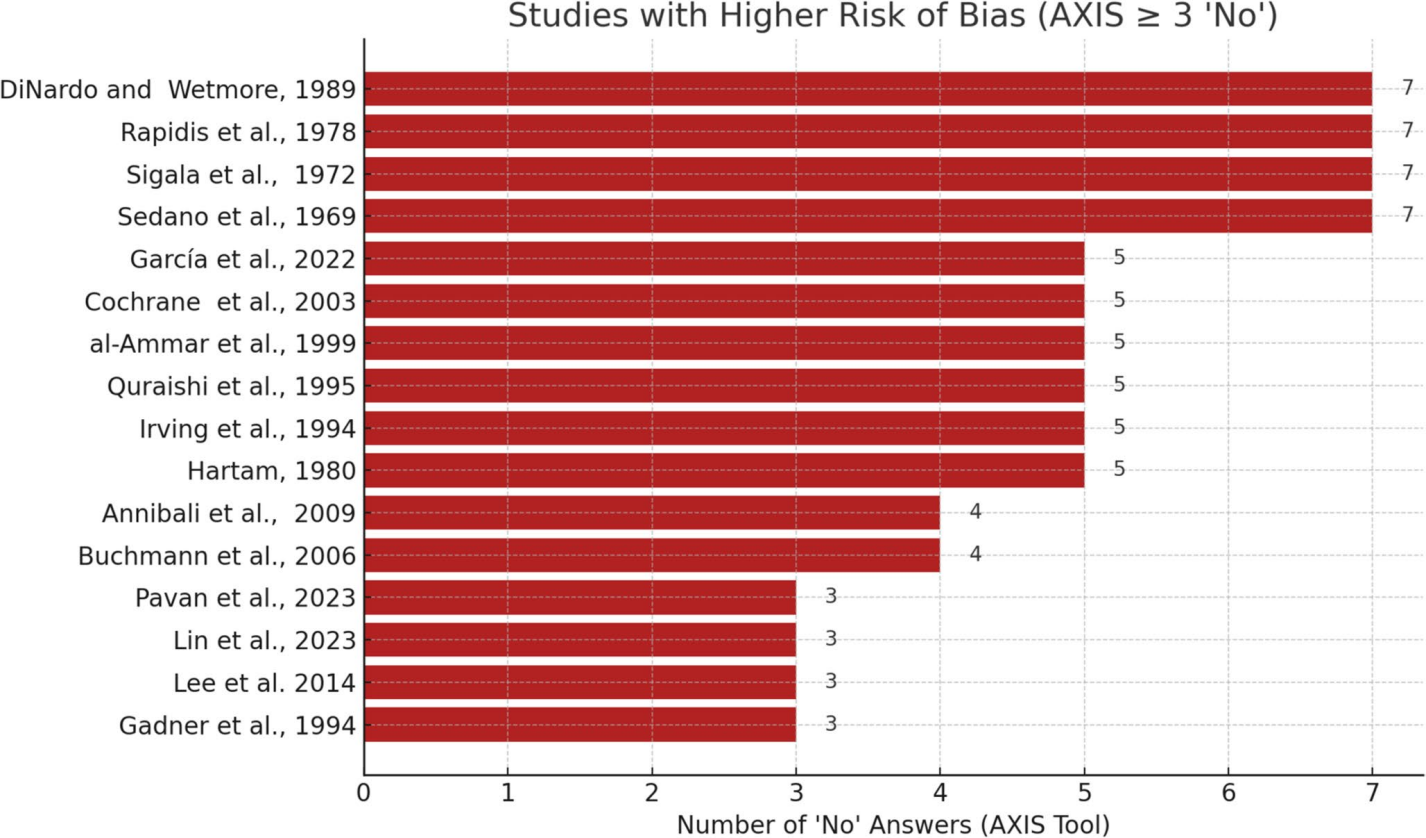
Studies with higher risk of bias according to the AXIS tool (≥ 3 ‘No’ Responses): Bar chart displaying the number of negative responses (“No”) per study, indicating potential methodological weaknesses

The methodological quality, assessed using the AXIS tool, was generally moderate. Common weaknesses included the lack of formal statistical analyses, incomplete or unspecified follow-up, and the absence of ethical approval. Nonetheless, almost all studies clearly described their objectives, target population, and clinical characteristics. More recent studies tended to report diagnostic criteria more accurately, whereas older studies often lacked ethical approval or statistical justification (Fig. 7).

The results are presented in Table 5, where two reviewers independently assessed 20 items, assigning one of the following ratings for each study: Yes, No, or Do not know.

**Table 5:** AXIS tool assessment of methodological quality in included studies on oral manifestations of LCH: synthesis of responses provided by two independent reviewers to the 20-item AXIS checklist. Answers are reported as ‘Yes’, ‘No’, or ‘Do not know’

Autor, Data	1. Were the aims/objectives of the study clear?	2. Was the study design appropriate for the stated aim(s)?	3. Was the sample size justified?	4. Was the target/reference population clearly defined?	5. Was the sample frame taken from an appropriate population base so that it closely represented the target/reference population under investigation?	6. Was the selection process likely to select subjects/participants that were representative of the target/reference population under investigation?	7. Were measures undertaken to address and categorise non-responders?	8. Were the risk factor and outcome variables measured appropriate to the aims of the study?	9. Were the risk factor and outcome variables measured correctly using instruments/measurements that had been trialled, piloted or published previously?	10. Is it clear what was used to determine statistical significance and/or precision estimates?	11. Were the methods (including statistical methods) sufficiently described to enable them to be repeated?	12. Were the basic data adequately described?	13. Does the response rate raise concerns about non-response bias?	14. If appropriate, was information about non-responders described?	15. Were the results internally consistent?	16. Were the results for the analyses described in the methods, presented?	17. Were the authors’ discussions and conclusions justified by the results?	18. Were the limitations of the study discussed?	19. Were there any funding sources or conflicts of interest that may affect the authors’ interpretation of the results?	20. Was ethical approval or consent of participants attained?
	Yes / No / Do not know	Yes / No / Do not know	Yes / No / Do not know	Yes / No / Do not know	Yes / No / Do not know	Yes / No / Do not know	Yes / No / Do not know	Yes / No / Do not know	Yes / No / Do not know	Yes / No / Do not know	Yes / No / Do not know	Yes / No / Do not know	Yes / No / Do not know	Yes / No / Do not know	Yes / No / Do not know	Yes / No / Do not know	Yes / No / Do not know	Yes / No / Do not know	Yes / No / Do not know	Yes / No / Do not know
Pavan et al., 2023 [47]	Y	Y	No	Y	Y	Y	No	Y	Y	Y	Y	Y	Do not know	No	Y	Y	Y	Y	Y	Y
Lin et al., 2023 [48]:	Y	Y	No	Y	Y	Y	No	Y	Y	Y	Y	Y	Do not know	No	Y	Y	Y	Y	Y	Y
García et al., 2022 [49]	Y	Y	No	Y	Y	Y	No	Y	Y	No	Y	Y	Do not know	No	Y	Y	Y	Y	Y	No
Lee et al. 2014 [50]	Y	Y	No	Y	Y	Y	No	Y	Y	Y	Y	Y	Do not know	No	Y	Y	Y	Y	Y	Y
Annibali et al., 2009 [51]	Y	Y	No	Y	Y	Y	No	Y	Y	Y	Y	Y	Do not know	No	Y	Y	Y	Y	Y	No
Buchmann et al., 2006 [52]	Y	Y	No	Y	Y	Y	No	Y	Y	No	Y	Y	Do not know	No	Y	Y	Y	Y	Y	Y
Cochrane et al., 2003 [20]	Y	Y	No	Y	Y	Y	No	Y	Y	No	Y	Y	Do not know	No	Y	Y	Y	Y	Y	No
al-Ammar et al., 1999 [53]	Y	Y	No	Y	Y	Y	No	Y	Y	No	Y	Y	Do not know	No	Y	Y	Y	Y	Y	No
Quraishi et al., 1995 [54]	Y	Y	No	Y	Y	Y	No	Y	Y	No	Y	Y	Do not know	No	Y	Y	Y	Y	Y	No
Irving et al., 1994 [55]	Y	Y	No	Y	Y	Y	No	Y	Y	No	Y	Y	Do not know	No	Y	Y	Y	Y	Y	No
Gadner et al., 1994 [56]	Y	Y	No	Y	Y	Y	Y	Y	Y	Y	Y	Y	No	Y	Y	Y	Y	Y	Y	No
DiNardo and Wetmore, 1989 [57]	Y	Y	No	Y	Y	Y	No	Y	Y	No	Y	Y	Do not know	No	Y	Y	Y	No	No	No
Hartam, 1980 [19]	Y	Y	No	Y	Y	Y	No	Y	Y	No	Y	Y	Do not know	No	Y	Y	Y	Y	Y	No
Rapidis et al., 1978 [58]	Y	Y	No	Y	Y	Y	No	Y	Y	No	Y	Y	Do not know	No	Y	Y	Y	No	No	No
Sigala et al., 1972 [59]	Y	Y	No	Y	Y	Y	No	Y	Y	No	Y	Y	Do not know	No	Y	Y	Y	No	No	No
Sedano et al., 1969 [18]	Y	Y	No	Y	Y	Y	No	Y	Y	No	Y	Y	Do not know	No	Y	Y	Y	No	No	No

### Meta-analysis and sensitivity analysis

The meta-analysis was conducted using two software packages: OpenMeta[Analyst] version 10, which generated forest plots to visualise aggregated results, and Review Manager 5.4, developed by the Cochrane Collaboration (Copenhagen, Denmark). These tools were employed to synthesise and analyse the pooled data from the included studies, providing a comprehensive overview of the evaluated outcomes.

The first meta-analysis focused on the prevalence of oral lesions (involving both mucosal and jawbone sites) among patients diagnosed with histiocytosis. This analysis included 16 studies: Pavan et al., 2023 [47], Lin et al., 2023 [48], García et al., 2022 [49], Lee et al., 2014 [50], Annibali et al., 2009 [51], Buchmann et al., 2006 [52], Cochrane et al., 2003 [20], al-Ammar et al., 1999 [53], Quraishi et al., 1995 [54], Irving et al., 1994 [55], Gadner et al., 1994 [56], DiNardo and Wetmore, 1989 [57], Hartam, 1980 [19], Rapidis et al., 1978 [58], Sigala et al., 1972 [59] and Sedano et al., 1969 [18].

**Fig. 8:**
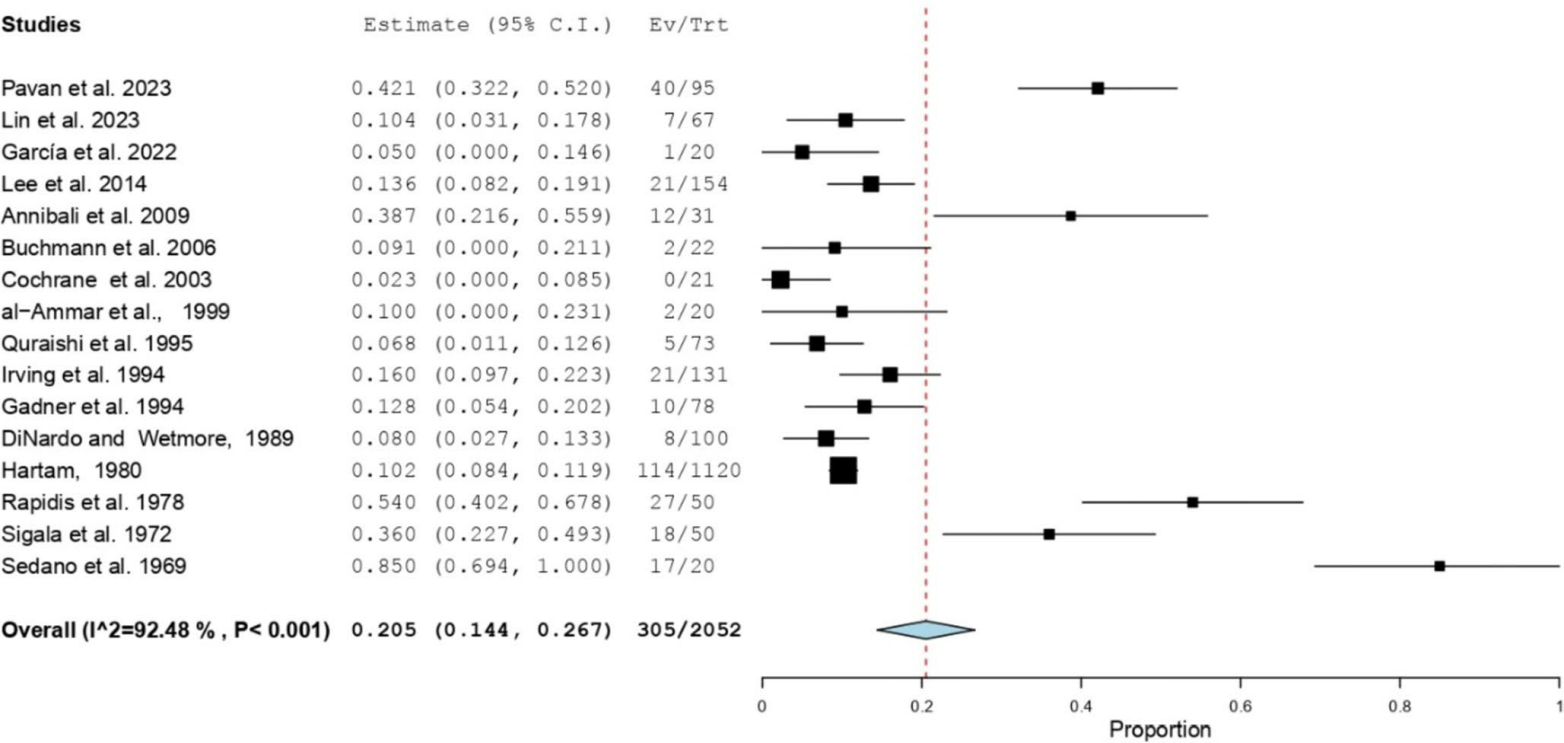
Binary random-effects model metric; proportion: 0.205; C.I. (Confidence Interval): (lower bound) 0.144(upper bound) 0.267; p-value < 0.001; P = p-value; heterogeneity (Het.): tau^2: 0.013; df = degrees of freedom; Q = Q statistic; Q (df = 15), 199.404 Het. p-value: < 0.001, I2: 92.478 ; Standard error (SE): 0.031; I^2^ (I^2) = Higgins heterogeneity index, I2 < 50%, heterogeneity low. Each study graphically presents the first author’s name and publication date, along with measurements such as the number of patients with oral lesions out of the total number of patients and their proportions, with their confidence intervals. The final summary value, highlighted in bold, also includes the corresponding confidence intervals. A red dotted line indicates the position of the mean value, while a blue diamond represents the mean effect measurement

A random-effects model, as described by DerSimonian and Laird, was applied to calculate the pooled ratio between the number of events (oral manifestations/oral lesions of histiocytosis) and the total number of patients diagnosed with LCH. The final result was 305 out of 2052 patients, corresponding to a prevalence of 14.86% (Fig. 8).

**Fig. 9:**
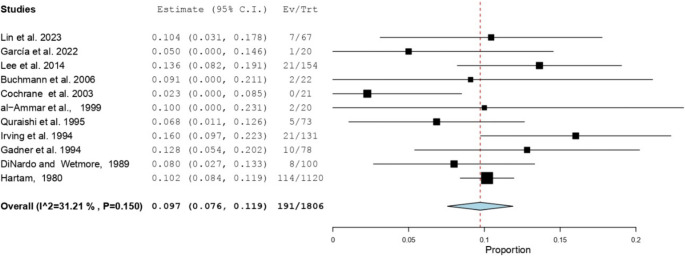
Sensitivity analysis. Binary random-effects model metric; proportion: 0.097; C.I. (Confidence Interval): (lower bound) 0.076(upper bound) 0.119; p-value < 0.001; P = p-value; heterogeneity (Het.): tau^2: 0.00001; df = degrees of freedom; Q = Q statistic; Q (df = 10), 14.538, Het. p-value: = 0.15, I^2^: 31.21; Standard error (SE): 0.011; I^2^ (I^2) = Higgins heterogeneity index

In addition, a sensitivity analysis was performed to identify potential sources of heterogeneity within the meta-analysis. Selective exclusion of studies with poor overlap of confidence intervals revealed that these studies were likely contributors to the observed heterogeneity (Pavan et al., 2023 [31]; Annibali et al., 2009 [35]; Rapidis et al., 1978 [58]; Sigala et al., 1972 [59]; and Sedano et al., 1969 [18]). Notably, the exclusion of these studies resulted in a substantial reduction in Higgins’ I² (inconsistency index), from 91% to 31% (Fig. 9).

The updated pooled prevalence following sensitivity analysis was 191 out of 1,806 patients, corresponding to 10.57**%** (Fig. 9).

### Publication bias, GRADE

**Fig. 10:**
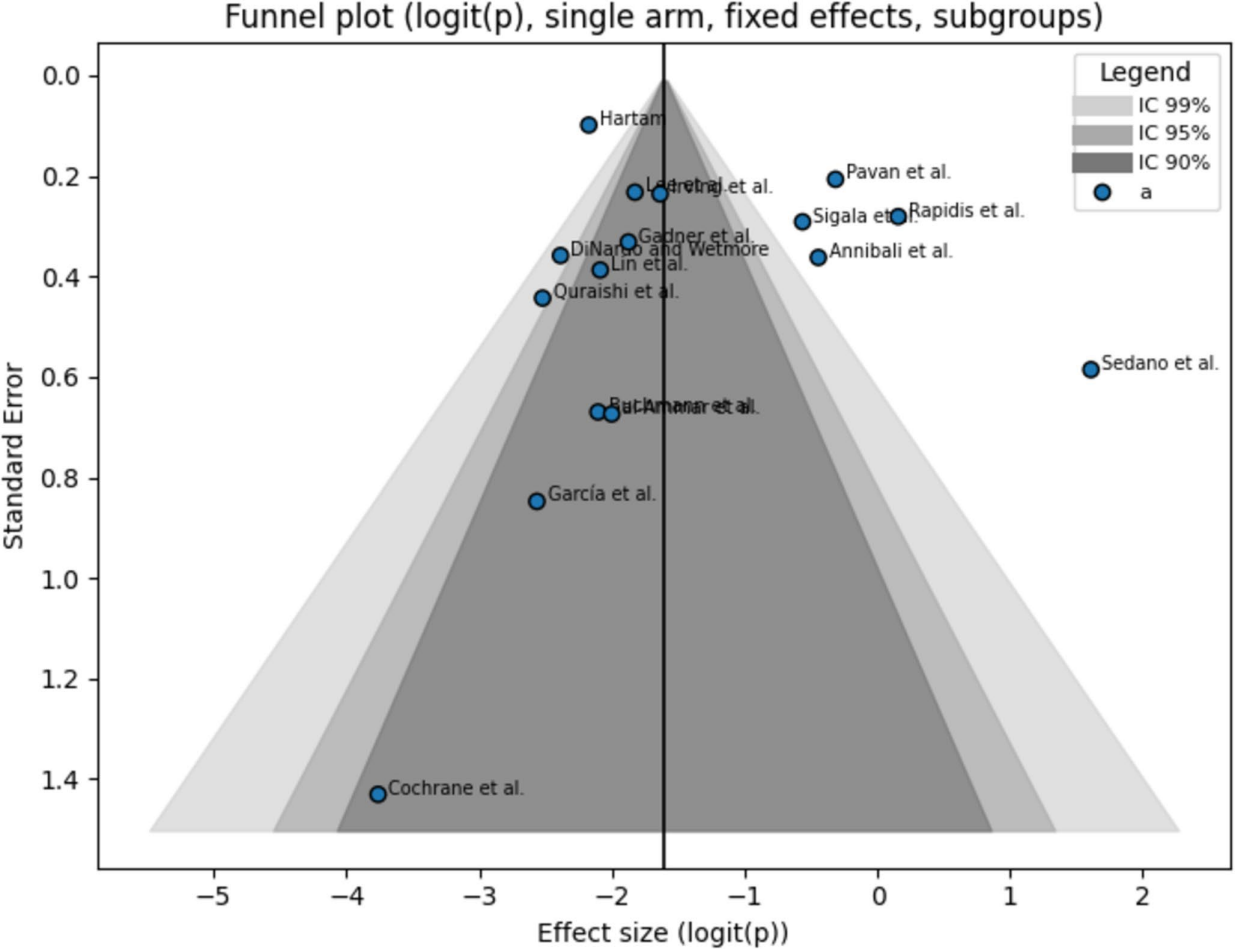
Funnel plot of included studies (Logit-transformed proportions, fixed effects model), assessment of publication bias based on logit-transformed proportions with continuity correction. Shaded areas represent 99%, 95%, and 90% confidence intervals; a: subgroup

Publication bias was assessed using a dedicated Python-based application previously developed by the authors [60]. Visual inspection of the funnel plot (Fig. 10) and Egger’s regression test were performed to evaluate potential asymmetry among studies. The funnel plot showed no marked asymmetry, and Egger’s test was not statistically significant (*p* = 0.3323), suggesting no strong evidence of publication bias (Table 6). The regression intercept was positive but associated with a wide confidence interval including zero, indicating limited robustness of the estimate. These findings are consistent with the overall visual symmetry of the funnel plot, with the possible exception of the study by Sedano et al. (1969) [18],, which appeared as a potential outlier. Nevertheless, these results should be interpreted with caution given the relatively small number of included studies and the substantial heterogeneity observed across studies, which may limit the ability of funnel plot–based methods to reliably detect publication bias.

**Table 6:** Results of Egger’s regression test for funnel plot asymmetry. Evaluation of small-study effects in the meta-analysis of oral lesion prevalence using logit-transformed proportions. Egger’s test was applied to assess the presence of small-study effects and potential publication bias. The test estimates the intercept (β₀) from a linear regression of the standardised effect size on its precision (1/SE). A statistically significant non-zero intercept suggests asymmetry in the funnel plot, which may indicate publication bias. In this analysis, the intercept was 1.5542 (*p* = 0.3323), suggesting no statistically significant evidence of asymmetry

Egger’s Test	Value
Intercept (β₀)	1.5542
Std Error	1.5476
t-value	1.0043
DF	14
p-value	0.3323
95% CI	[-1.4791, 4.5875]

The overall certainty of the evidence, as assessed using the GRADE approach, was rated as low, consistent with the observational and retrospective design of the included studies. Moderate heterogeneity was detected (I² ≈ 91.8%) and the precision of effect estimates varied across studies. However, no relevant concerns were identified regarding indirectness or significant publication bias Table 7.

**Table 7:** GRADE

GRADE Domain	Judgement	Notes
Risk of bias	Serious (retrospective, non-randomised studies)	All studies were observational and uncontrolled.
Inconsistency	Moderate (visible heterogeneity, I² ≈ 91.8%)	Estimated I² of approximately 91.8%; moderate heterogeneity observed.
Imprecision	Moderate (some studies with wide confidence intervals)	Precision varied between small and large studies.
Indirectness	None (clinically relevant outcome)	The outcome is directly applicable to the target population.
Publication bias	Not serious (Egger’s test not significant)	Funnel plot analysis showed no significant asymmetry.

## Discussion

The analysis of aggregated data extracted from studies with predominantly retrospective designs revealed that approximately 14.65% of patients diagnosed with histiocytosis present with oral manifestations at the time of diagnosis. These manifestations involve either the oral mucosa and/or the jaw bones. This figure, derived from 305 cases out of 2,082 patients, highlights the clinical importance of thorough oral cavity examinations during the diagnostic work-up of histiocytoses, particularly LCH. Given the young age at which some forms are typically diagnosed, such assessments should be coordinated with paediatricians.

Our findings align with previous reports suggesting that oral lesions may be among the earliest manifestations of LCH, and that in some cases the oral cavity may represent the only site of disease involvement. These lesions can also represent reactivations of the disease, as described by Sigala et al., 1972 [59]. It is worth noting that the included studies focused specifically on oral and maxillofacial manifestations potentially detectable by pediatric dentists or general dentists—particularly during panoramic radiographic screening—rather than the entire spectrum of head and neck involvement typically assessed by oral and maxillofacial surgeons.

The prevalence of oral lesions varied widely across studies (Figs. 4), ranging from 86% in the study by Sedano et al. (1969) [18] to 0% in Cochrane et al., 2003 [20] possibly reflecting different diagnostic protocols or referral patterns. A potential correlation between oral involvement and age at diagnosis was hypothesised, but data analysis, weighted for sample size, did not support a consistent relationship.

Lesions were predominantly located in the mandible, particularly in the ascending ramus and posterior body, and could be either solitary or multiple. Alveolar bone loss often led to a periodontal-like clinical presentation, with gingival recession, destruction of the keratinised gingiva, deep periodontal pocket formation, swelling, and pain. This process resulted in an osteolytic pattern associated with “floating teeth” due to the loss of periodontal ligament and exposed root surfaces.

Osteolysis and the associated periodontal inflammation may facilitate ectopic and premature eruption of the permanent molars. In children under one year of age, premature eruption of primary teeth can occur, and the loss of developing permanent tooth buds has been reported. Any disruption in the jawbones can have permanent sequelae on both primary and permanent dentition [61].

Radiographically, osteolytic lesions in the alveolar bone often appeared with flat bases and rounded borders, resembling perforations made by a punch tool. Some authors described oral mucosal lesions as ovoid, erythematous, and tender on palpation, mainly located in the vestibular sulcus and buccal mucosa. When such lesions affect the maxillary mucosa, the prognosis is more severe, as they may invade the orbit, maxillary sinus, and even reach the skull base, posing neurological risks [62].

In single-system LCH, oral lesions were limited to bone, while multisystem subtypes exhibited more diverse involvement, affecting bone, mucosa, or both. Moreover, the age at diagnosis varied significantly with disease presentation: 0–2 years for multisystem forms, and older than 2 years for localised or single-system lesions.

### Diagnostic considerations

From a diagnostic perspective, this review confirms the critical role of histological evaluation supported by immunohistochemical staining. Most of the included studies consistently highlight the use of CD1a and S-100 markers as the current gold standard for the identification of Langerhans cells. Although electron microscopy allows direct identification of Birbeck granules—cytoplasmic structures that are pathognomonic for Langerhans cell histiocytosis (LCH) and historically considered the definitive diagnostic tool—the use of CD207 (langerin) has demonstrated excellent diagnostic concordance. This marker binds to a transmembrane protein associated with Birbeck granules and represents a more practical alternative in routine diagnostic settings (Jalil and Hin-Lau, 2009 [45]).

In addition to these markers, CD68 has also been evaluated (Jalil and Hin-Lau, 2009 ). Although commonly used to identify cells of the monocyte/macrophage lineage, CD68 shows only weak expression in immature Langerhans cells, with a progressive decrease in expression as the cells mature. Therefore, while CD68 is useful for identifying macrophage components, it is not specific for the identification of Langherans cell, as previously emphasised by Jalil and Hin-Lau (2009) [45].

Bedran et al. (2018) investigated the aggressiveness of LCH by analysing the expression of the proliferation index Ki-67, which exceeded 20% in most patients, suggesting high biological activity and potentially more aggressive clinical behaviour. Histopathological findings in certain cases also revealed necrosis, apoptosis, eosinophilic infiltrates, and the presence of multinucleated giant cells, the latter often associated with tissue necrosis. Additionally, Bedran et al. (2018) explored a possible role of the Epstein–Barr virus (EBV), but all samples tested negative, excluding a direct viral association in the examined cohort [44].

From a differential diagnostic standpoint, LCH may resemble other osteolytic pathologies. Osteomyelitis, for instance, can present with similar inflammatory infiltrates but lacks the characteristic proliferation of Langerhans cells and eosinophilic microabscesses. Malignant bone tumours may radiographically mimic LCH but are histologically composed of uniform neoplastic cells without mixed inflammatory infiltrates and do not express CD1a or S-100 [63]. Conversely, lymphomas are typically positive for LCA, L26, and UCHL-1, while Ewing sarcoma is characteristically CD99-positive [28].

In conclusion, the diagnostic approach to LCH must be multidisciplinary, relying on the integration of clinical, radiological, histopathological, and immunophenotypic data. Only through such a comprehensive framework can timely and accurate diagnosis be achieved, which is crucial for appropriate clinical management.

### Summary of findings and characteristics of included studies

The analysis of the selected studies revealed substantial variability in both the frequency and type of oral manifestations associated with Langerhans cell histiocytosis (LCH), reflecting the clinical heterogeneity of the disease. Reported prevalence rates ranged from no oral involvement in some cohorts [20] to more than 50% of cases in others [56, 59]. Oral manifestations most commonly included osteolytic jaw lesions, gingival ulceration, mucosal lesions, periodontal destruction, tooth mobility, pain, and early tooth loss [51, 54, 59]. Several studies identified the mandible as the most frequently affected craniofacial site, although maxillary involvement was also commonly reported [19, 48, 55]. Furthermore, oral lesions were frequently described as among the earliest clinical manifestations of LCH and, in some cases, represented the presenting sign of the disease [47, 51]. Oral involvement was often associated with concomitant craniofacial or systemic lesions, particularly affecting the skull and other skeletal sites [19, 55]. Considerable differences in patient characteristics, disease extent, diagnostic criteria, and study design likely contributed to the heterogeneity observed across studies and to the wide variation in reported prevalence estimates.

In summary, the studies included in this review underscore the clinical heterogeneity of Langerhans cell histiocytosis and its variable oral involvement. While some studies documented a high prevalence of oral lesions, others reported minimal or no involvement, reflecting differences in study populations, diagnostic criteria, and disease stages. Despite this variability, the data consistently highlight the diagnostic value of oral signs, often among the earliest clinical manifestations of LCH, underscoring the importance of interdisciplinary collaboration between dental professionals and paediatric specialists for timely diagnosis and management.

### Clinical implications

Oral manifestations of LCH may represent the first clinical sign of the disease and, in some cases, may precede the diagnosis of systemic involvement [51, 64] Therefore, dentists, oral medicine specialists, and pediatricians play a crucial role in the early recognition of affected patients. The findings of the present review indicate that tooth mobility, alveolar bone loss, gingival swelling, oral ulcerations, and jaw osteolytic lesions are among the most frequently reported manifestations. These features may closely resemble more common oral conditions, including aggressive periodontal disease, odontogenic infections, reactive inflammatory lesions, and benign or malignant neoplasms, potentially leading to delayed diagnosis or inappropriate treatment [51, 59, 64]. Oral lesions may also occur during disease relapse and have been proposed as possible indicators of progression toward multisystem involvement [51]. Consequently, persistent or atypical oral lesions that do not respond to conventional therapy should raise clinical suspicion, particularly in pediatric patients and in individuals with a history of LCH. In such cases, histopathological evaluation supported by immunohistochemical analysis remains essential to establish a definitive diagnosis and to distinguish LCH from other inflammatory or neoplastic disorders of the oral cavity [65]. Early identification and multidisciplinary management involving dentists, pediatricians, pathologists, and medical specialists may facilitate timely diagnosis, improve patient monitoring, and optimize clinical outcomes.

### Limits of the review

This review presents several methodological limitations, primarily related to the retrospective design of most included studies, which may introduce selection and information bias, as highlighted by the risk of bias assessment using the AXIS Critical Appraisal Tool and the GRADE approach for evaluating the quality of evidence. As a consequence, the strength of the conclusions that can be drawn from the available evidence remains limited, and the pooled estimates should be interpreted with appropriate caution. Moreover, the considerable heterogeneity among studies—both in terms of patient characteristics and diagnostic or therapeutic methodologies—limits the generalisability and robustness of the conclusions. The inconsistency index (I²) was initially 91%, but dropped to 31% only after the exclusion of five studies (Pavan et al., 2023 [31]; Annibali et al., 2009 [51]; Rapidis et al., 1978 [58]; Sigala et al., 1972 [59]; and Sedano et al., 1969 [18]) as revealed by sensitivity analysis (Fig. 9). Notably, three of these studies were among the oldest included and also exhibited lower methodological quality in the risk of bias assessment (Fig. 7). Furthermore, these studies reported substantially higher prevalences of oral manifestations compared with more recent investigations, suggesting that differences in diagnostic criteria, disease classification, referral patterns, and reporting standards over time may have contributed to the observed heterogeneity. In addition, diagnostic approaches for LCH have evolved and become increasingly standardized over time, including the use of immunohistochemical markers such as CD1a, S100, and CD207 (Langerin) to support diagnostic confirmation. Although the diagnostic methods adopted by individual studies were not always fully reported, differences in diagnostic approaches may have influenced case ascertainment and should be considered when comparing prevalence estimates across different periods. Therefore, while the overall pooled estimate provides a broad overview of oral involvement in LCH, the prevalence obtained after sensitivity analysis may better reflect contemporary clinical practice. Another limitation lies in the relatively small number of included studies (*n* = 16), which may not fully represent the entire spectrum of oral manifestations in histiocytic disorders.

Future perspectives should priorities multicentric, prospective studies with standardised diagnostic criteria to further elucidate the clinical and pathogenic links between histiocytosis and oral involvement. The integration of advanced diagnostic technologies, such as three-dimensional imaging and molecular tissue analysis, may offer additional insights to enhance early detection and support a more personalised treatment approach.

## Conclusion

The primary outcome of this systematic review with meta-analysis was to determine the prevalence of oral manifestations in patients with histiocytosis, in particular Langerhans Cell Histiocytosis (LCH).

This review confirms that oral manifestations represent a relevant clinical component of histiocytosis, with an overall prevalence of approximately 14.65%. These manifestations, often underestimated, may constitute the initial presentation of the disease and predominantly involve the mandible, presenting as osteolytic and mucosal lesions. Accurate clinical and radiographic evaluation, supported by histological and immunohistochemical analysis, remains essential for an early and correct diagnosis. However, the high methodological heterogeneity of the studies and the low overall quality of the evidence highlight the need for further well-designed multicentre, prospective studies using standardized diagnostic criteria and reporting methodologies to optimize the diagnostic and therapeutic approach to these patients and to provide more reliable estimates of the prevalence and clinical significance of oral manifestations in LCH.

## Supplementary Information

Below is the link to the electronic supplementary material.

Supplementary Material 1

Supplementary Material 2

## Data Availability

No datasets were generated or analysed during the current study.
