# Supplementary material for: Prevalence of oral lesions in Langerhans cell histiocytosis: a systematic review and meta-analysis
Source: Oral Maxillofac Surg. 2026 Jul 3;30(1):106. doi: 10.1007/s10006-026-01586-w (PMC13331839; doi:10.1007/s10006-026-01586-w)
Supplement: Supplementary file 2 — Supplementary Material 2 [file 10006_2026_1586_MOESM2_ESM.docx]

**Supplementary Materials**

The main data and results of the excluded studies are reported below.

1. Neves-Silva et al. (2018) [30] reported that the most common oral manifestations were ulcerations and involvement of the hard palate mucosa, often associated with dental mobility, gingival bleeding, and recession. In fact, in eight out of nine patients, the diagnosis of LCH was prompted by the presence of oral manifestations. The study highlights how oral signs can represent the initial or even the sole clinical indicator of the disease.
2. McCaffrey and McDonald (1978) [24] described 22 cases of Histiocytosis X, emphasising the involvement of the temporal bone, which may lead to symptoms such as otalgia, chronic otorrhoea, and hearing loss. The authors discussed the use of surgery as a primary treatment in many of these cases.
3. Jones and Pillsbury (1984) [25] conducted a retrospective case series that provided one of the first comprehensive assessments of head and neck manifestations of LCH. The study underscored the importance of recognising early symptoms to avoid diagnostic delays and contributed to the development of differential diagnostic criteria with respect to other inflammatory or neoplastic diseases of the cervicofacial region.
4. Broadbent et al. (1989) [26], authored by the Clinical Writing Group of the Histiocyte Society, proposed a standardised approach for diagnosing and clinically evaluating children with LCH. Recommendations included mandatory tissue biopsy to confirm the presence of Langerhans cells. The report recognised the clinical heterogeneity of the disease, ranging from benign localised forms to potentially fatal systemic involvement, and offered a detailed guide for diagnosis and follow-up to improve inter-centre communication and support cooperative international studies.
5. The study by Hamre et al. (1997) [27], represents a large-scale analytical epidemiological investigation aimed at exploring possible risk factors for LCH in children. It focused on genetic predisposition, prenatal events, infections, neonatal onset, and previously reported immunological abnormalities. While the associations were deemed highly speculative, with reporting bias acknowledged as a limitation, a hypothesis was made regarding maternal lymphocyte transmission as a mechanism involved in LCH pathogenesis. Specifically, a higher incidence of urinary tract infections during pregnancy might facilitate the passage of maternal immune cells. Furthermore, an association was observed between early-life transfusions and increased risk. The lack of association with passive smoking suggested that intrauterine exposure to tobacco smoke is not a significant risk factor for childhood LCH.
6. Wang et al. (2010) [28] conducted a retrospective observational analysis on a cohort of 108 paediatric patients. Histological features were extensively investigated using immunohistochemical tissue biomarkers such as CD1a (100% positivity), S100 (90%), CD68 (41.7%), Lys (40%), and MAC387 (30%). Surgical treatment was predominant, and the five-year survival rate reached 98%.
7. The study by Modest et al. (2016) [29] is a retrospective review of medical records that identified 29 cases of temporal bone LCH from 20 patients. The report focused exclusively on otological manifestations (such as otorrhoea, otalgia, hearing loss, etc.) and did not provide any data on oral involvement.
8. Nicollas et al. (2010) [31] examined 42 patients, 31 of whom presented with head and neck localisation of the disease. Among these, 10 exhibited manifestations confined exclusively to the head and neck region.
9. The study by Dagenais et al. (1992) [32] is a retrospective investigation of the radiographic features of Histiocytosis X in the jaws. Only cases with radiographic evidence of the disease (29 in total) were included. The study described solitary intraosseous lesions of round or oval shape, frequently associated with new periosteal bone formation. Multiple lesions in the alveolar region, with well-defined margins and a "scooped-out" appearance (a zone of bone destruction preserving part of the alveolar ridge), represented another distinguishing feature. Some degree of sclerosis and mild root resorption were also observed. According to the authors, these radiographic features may assist in the differential diagnosis of Histiocytosis X in the jaws, particularly when biopsy and histopathological examination are inconclusive.
10. Mortellaro et al. (2006) [33] investigated eight cases, seven of which presented with oral manifestations involving both hard and soft tissues. The study emphasised that oral lesions may precede the onset of systemic symptoms and therefore serve as an early indicator of LCH. Patients may initially present with signs mimicking dental infections or periodontal disease resistant to conventional treatment, underscoring the importance for paediatricians and dentists to include LCH in the differential diagnosis. A definitive diagnosis requires histological confirmation and immunohistochemical analysis, particularly CD1a detection.
11. Hicks and Flaitz (2005) [34], in their review, highlighted the suspected neoplastic nature of LCH. The study was excluded from our analysis as it was a literature review, and neither the title nor the abstract clearly indicated original data on oral manifestations.
12. The study by Gadner et al. (2001) [35] was a prospective, multicentre, randomised clinical trial comparing two treatment regimens (vinblastine versus etoposide, both with initial corticosteroid administration) for multisystem Langerhans Cell Histiocytosis. A total of 143 patients were evaluated. The study was excluded as it did not report any data regarding oral manifestations. However, the authors highlighted that a rapid treatment response (within 6 weeks) might represent a new and strong prognostic factor, with lack of early response being associated with treatment failure and increased mortality, particularly in patients with risk organ involvement (liver, lungs, haematopoietic system, and spleen).
13. Bartnick et al. (2002) [36] retrospectively analysed medical records of 12 patients with LCH involving the oro-maxillofacial region. The lesions were mainly located in the posterior mandibular region and the anterior maxilla.
14. Ardekian et al. (1999) [37] reported that eosinophilic granuloma of the jaws predominantly affects young patients, with a strong predilection for mandibular localisation.
15. Howarth et al. (1999) [38] did not clearly specify the number of patients with oral manifestations, but rather reported the number of bone lesions, which may be multiple. Specifically, they described 23 mandibular lesions and 4 maxillary lesions (as shown in one of the figures). Additionally, seven cases with mucosal involvement were noted without precise localisation, along with one case of tooth mobility. The study also emphasised that patients with isolated bone LCH tend to have a favourable prognosis, whereas multisystem involvement—especially when the lungs or pituitary axis are affected—is linked to worse outcomes and an increased likelihood of recurrence.
16. The study by Slater and Swarm (1980) [39] was excluded as a detailed textual analysis revealed it to be a literature review focused on eosinophilic granuloma of bone (one of the three clinical forms of Histiocytosis X). This review analysed data from 227 publications. Among the noteworthy findings, the most common clinical presentation was pain, reported in 51% of cases, followed by swelling either in conjunction with pain (22%) or alone (21%).
17. Egeler and Nesbit (1995) [40] conducted a narrative literature review on Langerhans Cell Histiocytosis (LCH). As a review article, it did not present a defined cohort or consolidated patient dataset—no unique patient count nor specific prevalence of oral manifestations were provided. Rather, the authors synthesised findings from previously published studies. The review underscored that diagnosis requires the integration of histomorphological data (eosin and haematoxylin staining) with immunohistochemistry, using specific markers such as CD1a, S-100, and identification of Birbeck granules through electron microscopy.
18. Eckardt and Schultze (2003) [41] conducted a retrospective study focusing on oral histiocytosis cases. The study was excluded because it involved only 10 patients. Main conclusions included: prognosis varies with disease site and extent; surgical treatment is effective in localised forms; oral lesions in the mandible and maxilla can mimic periodontal infections; accurate diagnosis requires detailed radiographic assessment and immunohistochemical confirmation using CD1a and S-100.
19. Mínguez et al. (2003) [42] performed a retrospective study involving only 10 paediatric patients (aged 4 months to 3.2 years), leading to its exclusion. The authors found that all patients showed oral manifestations, often mimicking periodontal disease and potentially delaying diagnosis. In half the cases, these oral signs were the initial clinical presentation of the disease.
20. The study by Lewoczko et al. (2014) [43] was a retrospective investigation of head and neck manifestations of LCH in 88 paediatric patients, with a mean age at diagnosis of 4.4 years. It was excluded because it considered only cases with head and neck involvement. The study concluded that diagnosis is often preceded by recurrent otitis media episodes. Mortality in the cohort was 9.1%, a reduction compared to previous decades.
21. The study by Bedran et al. (2018) [44] was a multicentric retrospective analysis of paediatric Langerhans Cell Histiocytosis (LCH) cases with head and neck involvement, including 19 patients. It was excluded as it only considered cases with head and neck localisation. The main findings highlighted that, in some patients, especially those with periodontal involvement, oral manifestations were the first clinical sign of the disease. The role of Epstein–Barr Virus (EBV) was also investigated, but none of the cases tested positive, thereby excluding a viral association in this cohort. Additionally, the Ki-67 proliferation index was used to assess disease aggressiveness; it exceeded 20% in most patients, suggesting a clinically active form.
22. The study by Jalil and Hin-Lau (2009) [45] was a retrospective analysis of 17 paediatric cases of oral LCH, with a mean age at diagnosis of 2.8 years. It was excluded because only patients already presenting with oral manifestations were included. The study confirmed the diagnostic value of immunohistochemistry using CD1a and S-100 and suggested, where feasible, the use of more advanced diagnostic techniques such as identification of Birbeck granules by electron microscopy or the use of the langerin (CD207) antibody.
23. The study by Atarbashi Moghadam et al. (2015) [46] was excluded as cases of oral manifestation of LCH were retrospectively identified from histological specimens submitted to the Oral and Maxillofacial Pathology Department. This approach introduced a potential bias, as only LCH cases with oral presentation would be included. The study found a relative frequency of oral LCH of 0.34%, with the posterior mandible being the most common site. The mean age of patients was 27 years, with a clear male predominance. Most lesions were localised, and dental mobility was the most frequently observed oral symptom.
